# The complete chloroplast genome of the endangered plant *Paphiopedilum wardii* (Orchidaceae)

**DOI:** 10.1080/23802359.2018.1532352

**Published:** 2018-10-26

**Authors:** Lin Wang, Changhai Sui, Ying Wang, Jia Guo

**Affiliations:** a Jilin Engineering Vocational College, Siping City, Jilin Province, P.R. China;; b School of Food Production Technology and Biotechnology, Changchun Vocational Institute of Technology, Changchun City, Jilin Province, P.R. China;; c Agro-Biotechnology Research Institute, Jilin Academy of Agricultural Sciences, Changchun City, Jilin Province, P.R. China

**Keywords:** *Paphiopedilum wardii*, Illumina sequencing, chloroplast genome, MITObim

## Abstract

Chloroplast (cp) genome sequences provide a valuable source for phylogenetic analysis, which becomes a popular tool for population and phylogeny in a recent report. Here, the complete chloroplast genome of the *Paphiopedilum wardii* has been reconstructed from the whole-genome Illumina sequencing data. The circular genome is 169,820 bp in size and comprises a pair of inverted repeat (IR) regions of 33,641 bp each, a large single-copy (LSC) region of 98,872 bp and a small single-copy (SSC) region of 3666 bp. The total GC content is 33.8%, while the corresponding values of the IR regions, LSC, and SSC and are 38.7%, 31.7% and 26.7%, respectively. The chloroplast genome contains 151 genes, including 91 protein-coding genes, eight ribosomal RNA genes and 52 transfer RNA genes. Two genes (*ndhA* and *ndhB*) are pseudogenized or lost in its cpDNA. The maximum-likelihood phylogenetic analysis showed a close relationship with *P. armeniacum* in Orchidaceae. Our findings provide useful information for phylogenetic and evolutionary research of *Paphiopedilum* species.

The chloroplast is a plant-specific organelle that proceeds with photosynthesis and crucial biosynthesis such as synthesizing starch, fatty acids and other proteins relative to its special functions (Saski et al. 2007; Ravi et al. 2008). Since the chloroplast genome sequence is a reliable tool for phylogenetic and evolutionary research, the chloroplast genome of many valuable plants has recently been reported (Chen et al. 2018). *Paphiopedilum wardii* (Orchidaceae) is a rare species, which native to southwest of China and northern Myanmar. *P. wardii* has been widely cultivated in Europe as ornamental plant for its special and colourful flower. To facilitate its genetic research and contribute to its utilization, in this study, we assembled its chloroplast genome using high-throughput Illumina sequencing technology and analysed its phylogenetic evolution, which will be helpful for better understanding of evolution within the Orchidaceae and further studies on its chloroplast genetic engineering.

DNA samples were extracted from the fresh leaves that were collected from a single individual of *P. wardii* in Kunming, Yunnan Province and stored in our laboratory. The whole genome shotgun sequencing of *P. wardii* was performed by Biomarker Technologies Co, Ltd. (Beijing, China) using the Illumina HiSeq 2500 platform (Illumina, Hayward, CA). Total 25.3 M 125 bp raw reads were retrieved and trimmed by CLC Genomics Workbench v8.0 (CLC Bio, Aarhus, Denmark). A subset of 18.2 M trimmed reads were used for reconstructing the chloroplast genome by MITObim v1.8 (Hahn et al. 2013), with that of its congener *Paphiopedilum niveum* (GenBank: NC_026776.1) as the initial reference genome. A total of 19,314,256 individual chloroplast reads yielded an average coverage of 539.7-fold. The chloroplast genome was annotated in GENEIOUS R11 (Biomatters Ltd., Auckland, New Zealand) and was drawn to the circular chloroplast genome sequence map of OGDRAW 1.1.

The chloroplast genome of *P. wardii* is a double-stranded circular DNA molecule with 169,820 bp in size (MH191341). It comprises a pair of inverted repeat (IR) regions of 33,641 bp each, separated by a large single-copy (LSC) region of 98,872 bp and a small single-copy (SSC) region of 3,666 bp. The total GC content is 33.8%, while the corresponding values of the IR, LSC, and SSC region are 38.7%, 31.7% and 26.7%, respectively.

This chloroplast genome harbours 151 functional genes, including 91 protein-coding genes (PCGs), 52 tRNA genes and eight rRNA genes. 39 PCGs, 29 tRNA genes and 4 rRNA genes are located in the forward strand, while others are located in the reverse strand. Moreover, 18 genes contain one intron, while genes *ycf2*, and *ycf3* harbour two introns; all the other genes are intronless. Among them, 37 genes are involved in photosynthesis, seven genes are in substance metabolism and 32 genes are related with self-replication. Two genes *(ndhA and ndhB)* are pseudogenized or lost in its cpDNA when compared with that of a closed species, *P. dianthum* and *P. armeniacum* (Liu et al. 2006; Hou et al. 2017).

The maximum-likelihood phylogenetic tree was generated using 41 shared PCGs among 37 chloroplast sequences in Orchidaceae by MEGA 6.0 (Tamura et al. 2013), which showed the position of *P. wardii* was situated as the sister of *P. armeniacum* in Orchidaceae (Figure 1). Our findings will provide a foundation for further investigation of chloroplast genome evolution in *Paphiopedilum*.

**Figure 1.**
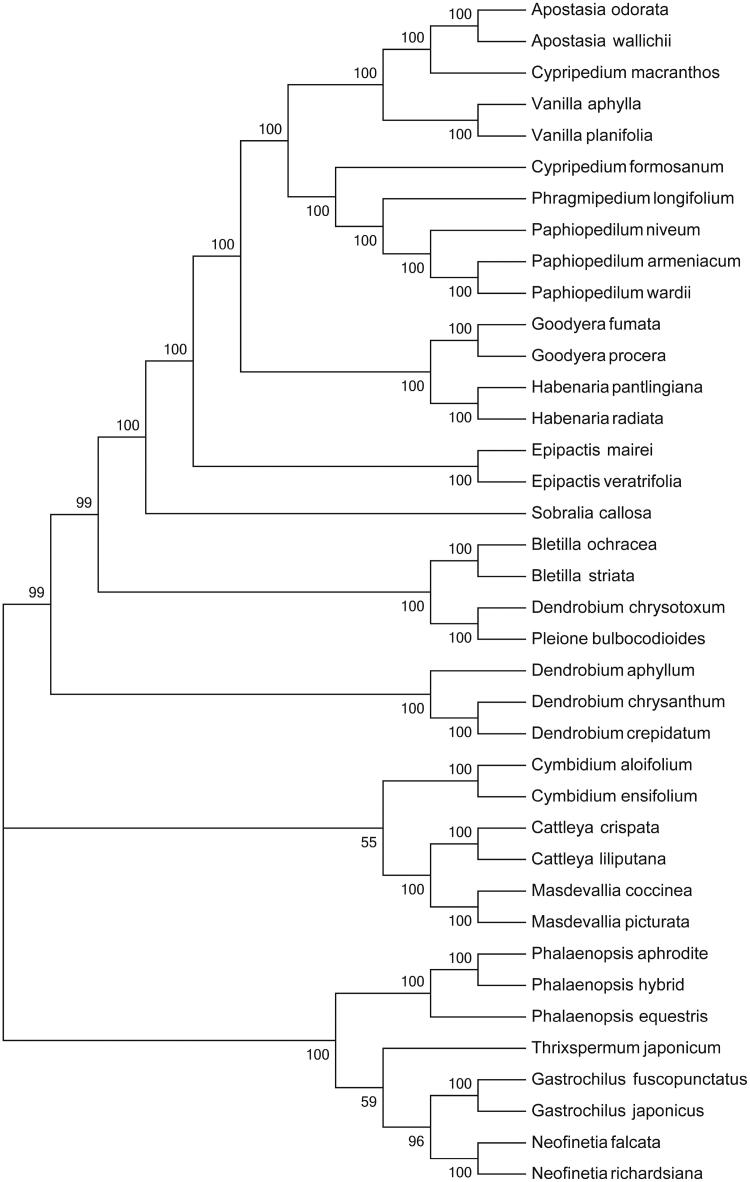
Phylogenetic of 38 species within the family Orchidaceae based on the maximum-likelihood analysis of the whole cp genome sequences using 500 bootstrap replicates. The analysed species and corresponding GenBank accession numbers are as follows: Apostasia odorata (NC_030722.1), Apostasia wallichii (NC_036260.1), Bletilla ochracea (NC_029483.1), Bletilla striata (NC_028422.1), Cattleya crispata (NC_026568.1), Cattleya liliputana (NC_032083.1), Cymbidium aloifolium (NC_021429.1), Cymbidium ensifolium (NC_028525.1), Cypripedium formosanum (NC_026772.1), Cypripedium macranthos (NC_024421.1), Dendrobium aphyllum (NC_035322.1), Dendrobium chrysanthum (NC_035336.1), Dendrobium crepidatum (NC_035331.1), Epipactis mairei (NC_030705.1), Epipactis veratrifolia (NC_030708.1), Gastrochilus fuscopunctatus (NC_035830.1), Gastrochilus japonicus (NC_035833.1), Goodyera fumata (NC_026773.1), Goodyera procera (NC_029363.1), Habenaria pantlingiana (NC_026775.1), Habenaria radiata (NC_035834.1), Masdevallia coccinea (NC_026541.1), Masdevallia picturata (NC_026777.1), Neofinetia falcata (NC_036372.1), Neofinetia richardsiana (NC_036373.1), Paphiopedilum armeniacum (NC_026779.1), Paphiopedilum niveum (NC_026776.1), Phalaenopsis equestris (NC_017609.1), Phalaenopsis hybrid cultivar (NC_025593.1), Phragmipedium longifolium (NC_028149.1), Pleione bulbocodioides (NC_036342.1), Sobralia callosa (NC_028147.1), Thrixspermum japonicum (NC_035831.1), Vanilla aphylla (NC_035320.1), Vanilla planifolia (NC_026778.1).
